# Transplanted sagebrush “wildlings” exhibit higher survival than greenhouse-grown tubelings yet both recruit new plants

**DOI:** 10.1186/s12862-024-02236-z

**Published:** 2024-04-22

**Authors:** Elizabeth C. Bailey, Eric Thacker, Thomas A. Monaco, Kari E. Veblen

**Affiliations:** 1 Dept. of Wildland Resources, Utah State University, 5230 Old Main Hill, Logan, UT 84322 USA; 2 Ecology Center, Utah State University, 5205 Old Main Hill, Logan, UT 84322 USA; 3 SWCA Environmental Consultants, 7210 Placid St, Las Vegas, NV 89119 USA; 4 U.S. Department of Agriculture, Agricultural Research Service, Forage and Range Research Laboratory, Utah State University, Logan, UT 84322 USA

**Keywords:** Bare-root, Containerized stock, Nucleation, Plugs, Rangeland, Rehabilitation, Restoration, Seedling, Transplant, Wilding

## Abstract

**Background:**

Land uses such as crop production, livestock grazing, mining, and urban development have contributed to degradation of drylands worldwide. Loss of big sagebrush (*Artemisia tridentata*) on disturbed drylands across the western U.S. has prompted massive efforts to re-establish this foundational species. There has been growing interest in avoiding the severe limitations experienced by plants at the seed and seedling stages by instead establishing plants from containerized greenhouse seedlings (“tubelings”). In some settings, a potential alternative approach is to transplant larger locally-collected plants (“wildlings”). We compared the establishment of mountain big sagebrush (*A. tridentata* ssp. *vaseyana*) from tubelings vs. wildlings in southeastern Idaho. A mix of native and non-native grass and forb species was drill-seeded in a pasture previously dominated by the introduced forage grass, smooth brome (*Bromus inermis*). We then established 80 m x 80 m treatment plots and planted sagebrush tubelings (*n* = 12 plots, 1200 plants) and wildlings (*n* = 12 plots, 1200 plants). We also established seeded plots (*n* = 12) and untreated control plots (*n* = 6) for long-term comparison. We tracked project expenses in order to calculate costs of using tubelings vs. wildlings as modified by probability of success.

**Results:**

There was high (79%) tubeling and low (10%) wildling mortality within the first year. Three years post-planting, chance of survival for wildlings was significantly higher than that of tubelings (85% and 14% respectively). Despite high up-front costs of planting wildlings, high survival rates resulted in their being < 50% of the cost of tubelings on a per-surviving plant basis. Additionally, by the third year post-planting 34% of surviving tubelings and 95% of surviving wildlings showed evidence of reproduction (presence / absence of flowering stems), and the two types of plantings recruited new plants via seed (3.7 and 2.4 plants, respectively, per surviving tubeling/wildling).

**Conclusions:**

Our results indicate that larger plants with more developed root systems (wildlings) may be a promising avenue for increasing early establishment rates of sagebrush plants in restoration settings. Our results also illustrate the potential for tubelings and wildlings to improve restoration outcomes by “nucleating” the landscape via recruitment of new plants during ideal climate conditions.

**Supplementary Information:**

The online version contains supplementary material available at 10.1186/s12862-024-02236-z.

## Background

The extent of intact drylands worldwide has been reduced by land uses, threatening the environment and ecosystem services upon which 38% of the global population rely [1–5]. The condition of these drylands is further threatened by factors such as non-native species invasions, woody plant encroachment, and the effects of climate change including increased aridity and temperature and more severe fire regimes [4–6]. A complementary strategy for stemming the loss of “wild” drylands is to expand restoration efforts to include rehabilitation of former agricultural areas [7, 8]. Regardless, dryland restoration of any kind increasingly requires new and innovative restoration practices as traditional methods often fail to meet restoration goals [9, 10].

Although direct seeding of desirable plant species is considered the most economically feasible approach for broadscale restoration efforts in drylands [10, 11], recent global reviews reveal increasing interest across a variety of ecosystems in the alternative practice of planting seedlings [12, 13]. Because germination and emergence are widely recognized as the life stages most limiting to establishing plants from seed in drylands [14–18], planting seedlings, often greenhouse-grown, serves as a strategy to bypass the biotic and abiotic limitations at these early life stages. The few comparative studies in drylands indicate higher early establishment and survival of planted seedlings than plants established from seed [11–13, 19], though the costs of growing and planting seedlings often are deemed to outweigh the benefits of their higher survival [11, 20]. However, in the face of prolonged and more frequent droughts, investment in transplanted seedlings– if they survive at sufficiently high rates– may be increasingly economically sensible and necessary to meet restoration goals.

Restoration is a major priority in big sagebrush ecosystems of the western U.S., which once covered 63 million hectares but have been reduced to half of their historic extent [6, 21]. Attempts to establish the foundational shrub species, big sagebrush (*Artemisia tridentata* Nutt.), from seed have been costly and largely unsuccessful [22–24] (but see [25]), attributable to an array of factors: short seed longevity, limited soil moisture, high interannual weather variability, maladapted seed sources, and resource competition with both native and non-native species [26–28]. The potential benefits of bypassing the vulnerable seed-to-seedling stage has driven a growing interest in planting containerized greenhouse seedlings (“tubelings”) of big sagebrush [24, 29]. Although they require greater upfront investment (i.e., soil, containers, seed, greenhouse space, water, labor for watering, transportation and planting), tubelings of big sagebrush can yield high survival rates (e.g., up to 50–89% 3-year survival) [30–32]. Due to these potentially high survival rates, use of tubelings has been recommended as a complementary strategy to offset the unreliable establishment associated with direct seeding [33].

A little-explored alternative to the use of big sagebrush tubelings is the transplantation of locally harvested “wildlings”, which we define here as larger, established plants harvested from nearby stands and transplanted with an intact soil-root ball. Larger-sized plantings of woody species have the advantage of more and better developed root systems [34] and often survive better than smaller plant stock [35]. Survival probability of established sagebrush plants also increases with plant size, and population growth rates are limited by transitions from small to large size classes [36], which together suggests that larger transplants could speed long-term population recovery. A handful of studies have investigated transplanting larger big sagebrush plants as “bare root” stock (i.e., without a soil-root ball), an approach which can leave plants vulnerable to transplant shock and damage during transplantation, and result in variable survival [34, 37, 38]. Though the logistics of harvesting, planting, and transporting wildlings with a soil-root ball intact can be costly, wildlings of woody species in a few studies have exhibited high (e.g., 80–100%) survival in big sagebrush systems [39–41]. We found one direct comparison of big sagebrush tubelings and wildlings that revealed no clear advantage of either approach for establishment of the *wyomingensis* subspecies [42], and we found no direct comparisons for the *vaseyana* subspecies that occurs in wetter sites and establishes at higher densities. Both tubelings and wildlings have generally been considered cost-prohibitive for land managers due to their upfront cost, but considering the cost per established plant (i.e., “cost as modified by the probability of success” sensu [43, 44]), is necessary for evaluating the efficacy of the different methods.

The objective of this study was to directly compare short-term survival of tubelings and wildlings of mountain big sagebrush (*A. tridentata* ssp. *vaseyana*) in the context of rehabilitation of former dryland pastures, as well as compare costs between the two methods. We also established broadcast seeding plots as basis for longer-term comparisons (of non-survival metrics) since seeding is commonly used by managers for landscape-scale restoration projects in the region.

## Methods

### Study area

This research was conducted on the 1605-ha Fox Hills Ranch, owned by Bayer since 2008, in Caribou County, ID, USA (42.769415, -111.472490) (Fig. S1). The majority of precipitation falls as snow from November to January, and there is a pronounced summer dry period from June to August. At a SNOTEL weather station 20 km from the project site (Station ID USS0011G01S; Somsen Ranch [45]) mean annual precipitation from 1991 to 2020 was 693 ± 19 mm. Annual precipitation for the four years encompassing our study (2019–2023) was 780 mm, 642 mm, 716 mm, and 602 mm (Table S1). Soils are generally loamy and well-drained [46], and native vegetation communities on the ranch include aspen (*Populus tremuloides* Michx.) groves, riparian areas, and big sagebrush plant communities. Prior to 2008, pastures were seeded with non-native grasses to enhance livestock forage production. The entire 1605 ha of Fox Hills Ranch supports an average of 2600 AUMs of cattle annually and is grazed early summer through mid to late October.

Our 96-ha project area ranged from 1926 to 1966 m in elevation and was comprised of two fenced pastures that were part of the regular cattle grazing rotation until the start of the project. The pastures were dominated by the non-native perennial grasses smooth brome (*Bromus inermis* Leyss.) and Kentucky bluegrass (*Poa pratensis* L.). Both are sod-forming grasses that have been widely planted across the United States for livestock forage. Their dominance and suppression of native species establishment and succession are now of major concern to land managers [47]. There were scattered mountain big sagebrush (*A. tridentata* ssp. *vaseyana* [Rydb.] Beetle) plants throughout the project area with higher densities along the southern and western boundaries.

### Site preparation

The 96-ha project area was grazed by cattle in September and October 2018 to reduce aboveground biomass of introduced perennial grasses. Grazing was then suspended for the remaining duration of the project, and the fence separating the two pastures was removed. In October of 2018 the entire project area was disced (three passes; Case IH Ecolo Tiger 870 22’ Disc Ripper) and received a final pass with a harrow (60’ 15 Bar McFarlane Harrow Cart). An herbicide mixture of glyphosate (Roundup Power Max, Monsanto Company, St. Louis, MO) and dicamba (Vision, Helena Agri-Enterprises, LLC) was applied with a boom sprayer (Case SPX 4430) at rate of 2.33 L / hectare and 0.15 L / hectare respectively, on July 31st, 2019 to reduce cover of the non-native forage grasses. On the 24th and 25th of September, 2019, final seedbed preparation was completed in the project area with one pass of a chisel plow and one pass with a harrow, leaving the site almost entirely as bare soil except for approx. 5–10 remnant sagebrush individuals in the southeast portion of the study area.

In October 2019, the project area was drill-seeded with a seed mix comprised of twenty-two rangeland species that included eight perennial grasses and fourteen perennial forbs, the majority of which were selected to provide cover and support the dietary needs of the Greater Sage-Grouse (*Centrocercus urophasianus*) (Table S2). Seed was purchased from ACF West, Boise, ID, which is supplied by Clearwater Seed (https://www.clearwaterseed.com/) Spokane, WA, a reputable supplier of Certified and Source Identified seed in adherence with the Association of Official Seed Certifying Agencies (AOSCA; https://aosca.org/) standards. Of the 96 ha in the project area, 86 ha were seeded at a rate of 14.4 kg pure live seed (PLS) / ha with a precision drill seeder (Truax OTG 7518G, New Hope, MN) with 18 opener discs (19.05 cm apart) at a depth between 0.95 cm and 1.27 cm. Due to several early season storms, 8 ha were inaccessible by the drill seeder and were instead broadcast seeded using a Brillion seeder at double the original seeding rate (28 kg PLS / ha) to compensate for reduced efficacy of broadcast vs. drill-seeding. The remaining 2 ha were on a steep slope and inaccessible due to wet conditions and remained unseeded.

### Experimental design

**Fig. 1:**
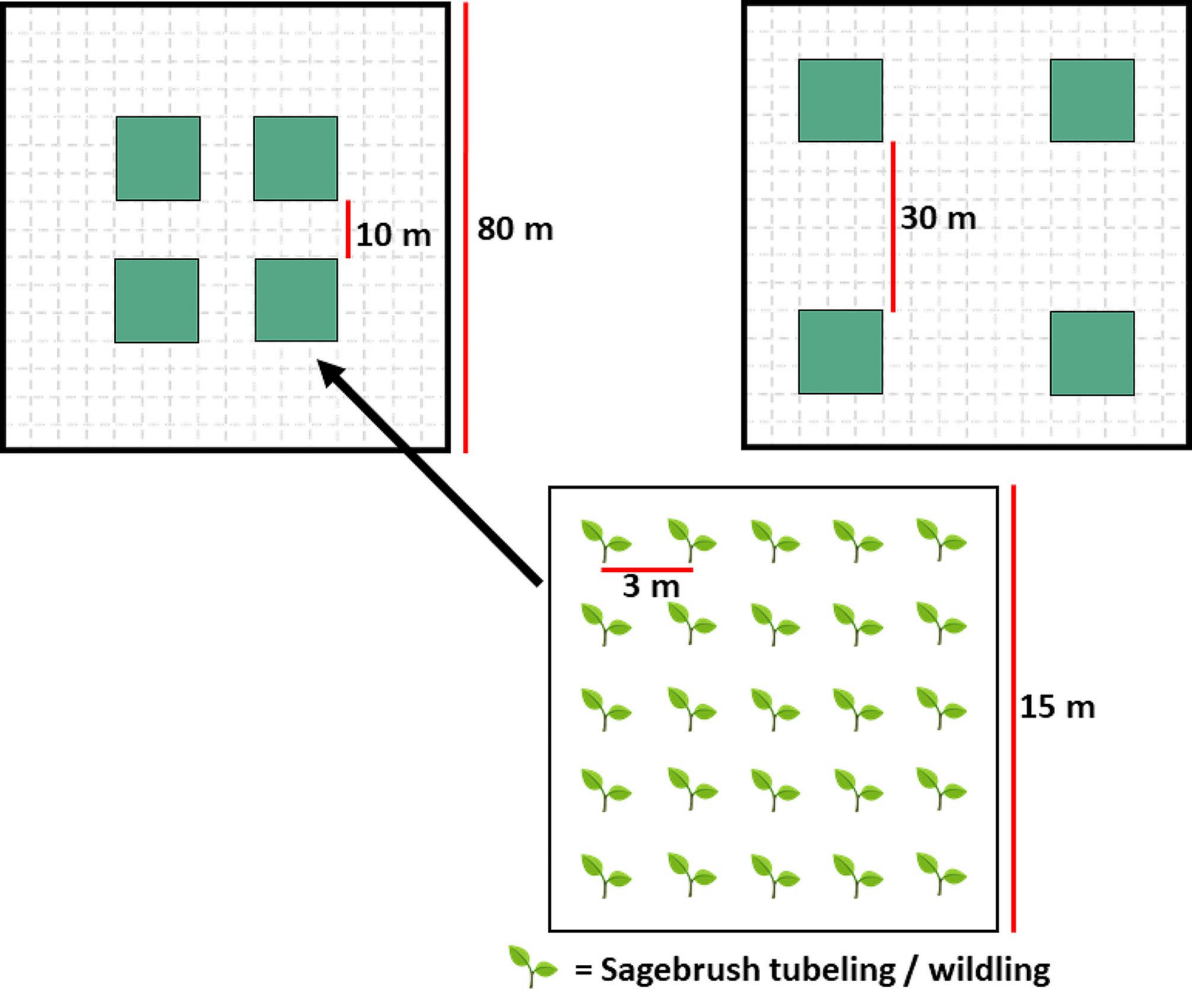
Layout of experimental plots that were used to compare sagebrush establishment via tubeling, wildling, and direct seeding treatments. Each 80 m x 80 m experimental plot contained four 15 m x 15 m “islands”, with islands located either 10 or 30 m apart. Within each island twenty-five *Artemisia tridentata* spp. *vaseyana* plants were planted on a 3 m-spaced grid for tubeling and wildling treatments. For the seeding treatment, the entirety of the four 15 m x 15 m islands were broadcast seeded. The 10 m vs. 30 m spacing of islands within plots is part of a different long-term study investigating the effects of island density on nucleation of sagebrush plant communities (sensu [76])

We established forty-two 80 m x 80 m plots in the 86ha that had been drill seeded, with a buffer of ≥ 15 m between plots, avoiding major landscape features such as washes. Thirty-six of these plots were randomly assigned to, and equally divided among, one of three big sagebrush (hereafter sagebrush) establishment methods that were applied in October/November 2019: tubelings, wildlings, and seeding (the latter established as a basis for comparison over the long term for non-survival metrics such as plant densities). The remaining six 80 m x 80 m plots were designated as untreated controls (i.e., no sagebrush establishment method) (Fig. S1). In any plot assigned to one of the three sagebrush establishment methods, the treatment was applied to four 15 m x 15 m sagebrush “islands” within the larger 80 m x 80 m plot (Fig. 1). In tubeling and wildling plots, each island contained twenty-five mountain sagebrush individuals planted in a 5 × 5 grid, with plants spaced 3 m apart (i.e., 100 total plants per plot; Fig. 1). For the seeding plots, each island was broadcast seeded by hand with sagebrush seed at a rate of 0.50 kg of PLS per ha, which is the highest density recommended to establish a sagebrush stand [48, 49]. Planting of seedlings and wildlings occurred between October 15th and November 11th, 2019. Seeding occurred October 24th through November 11th, 2019.

### Plant materials and planting technique

Sagebrush tubelings were grown by North Fork Native Plants (Rexburg, ID) with mountain big sagebrush seed purchased from Utah Seed (https://www.utahseed.com/) in Tremonton, UT, a reputable supplier of Certified and Source Identified seed in adherence with the Association of Official Seed Certifying Agencies (AOSCA; https://aosca.org/) standards. Seed had been collected in fall of 2018 near Logan, Utah, approximately 136 km from the project area, in a similar elevation and climate regime (41.557240, -111.808770). Sagebrush seed was sown into 164 ml³ containers (Ray Leach SC10 Cone-tainers, Steuwe & Sons, Inc, Tangent, OR) from July 1st to July 11th, 2019, with containers reseeded if no seedlings emerged. Plants were delivered to the project site on October 14th, 2019 and remained outdoors until the end of planting. For planting, dibble sticks were used to create holes, and tubelings were placed in holes by a contracted environmental consulting and habitat restoration crew who was instructed to subsequently firm the surrounding soil. No supplemental water was applied.

Sagebrush wildlings were harvested from private property (Caldwell Canyon) approximately 11 km from Fox Hills Ranch (42.722228, -111.35985) in an area slated for future vegetation removal associated with mining. These wild-collected mountain big sagebrush (*A. tridentata* ssp. *vaseyana*) plants were identified by Jeff Klausmann, owner of Intermountain Aquatics (https://www.intermountainaquatics.com/), a reputable habitat restoration consulting firm, and there are no local or national restrictions on collection of this species on private property. Habitat restoration crew members used round point digging shovels to dig up small (6–25 cm tall) non-reproductive sagebrush plants and a shovel-blade-sized amount of dirt surrounding the root ball (Fig. S2). The plants were wrapped in burlap and secured with twine, transported to the field site, and planted the same day as collected. For planting, a shovel was used to create holes, wildlings were removed from the burlap and placed inside the hole, and soil was compressed around each plant. No supplemental water was applied.

For the seeded treatment plots, islands were divided into five rows (3 m wide) and crew members were given a cup of seed filled with the amount to yield a seeding rate of 0.56 kg PLS per ha. Crew members then hand-scattered the seed evenly across each row and used a push roller (approximately 46 cm diameter x 61 cm long) to increase seed to soil contact. We used the same seed as was used for growing tubelings, and no supplemental water was applied.

### Data collection

Within five weeks of our October/November 2019 planting the following measurements were taken on all tubelings (*n* = 1200) and a subset of wildlings (*n* = 297; winter weather prevented us from measuring all wildlings): height (nearest cm from soil surface to tip of highest point of plant canopy), and crown size (longest axis and perpendicular axis in cm). We also made assessments of whether plantings had experienced frost heaving (yes / no), or showed obvious signs of planting problems (presence / absence of exposed roots or air pockets) or physical damage (presence / absence). Additionally, we noted when a tubeling or wildling had been transplanted with more than one plant growing in the container/rootball; for tubelings we retained one randomly-selected plant per microsite and clipped the others.

In the summer and fall following planting (June and October 2020, respectively) and in the summer three years post-planting (July 2022) we made the following assessments: (1) survival status (live / dead) of all planted tubelings and wildlings and (2) reproduction (presence / absence of flowering stems) of all live tubelings and wildlings. We measured height and crown size on all live wildlings in the summer (June 2020) following planting (because we had only done so for a subset immediately following the fall planting), and then measured height and crown size of all surviving tubelings and wildlings in the third-year post-planting (July 2022). We also recorded insect damage (galls, aphids, etc.), rodent damage, and evidence of large herbivore browsing for tubelings and wildlings during multiple sample periods.

We assessed sagebrush densities by size class in all tubeling (*n* = 12), wildling (*n* = 12), seeded (*n* = 12) and control (*n* = 6) plots in July/August 2022. In tubeling and wildling plots, our objective was to quantify new sagebrush that had recruited from seed produced by surviving tubeling and wildling plantings over the previous three years. To accomplish this, the belt in each island ran parallel to, and equidistant between, the center planting row and an adjacent planting row (to ensure the originally planted sagebrush were not included in our counts). Sagebrush plants were recorded by size class (< 15 cm, 15–50 cm, and > 50 cm) within one 1 m x 10 m belt in each island (i.e., 4 belts and 40 m^2^ per treatment plot). In seeded and control plots, our objective was to evaluate the outcome of the seeding treatment (and non-treatment in control plots). In these plots, belts were located in the same relative location and orientation as in the tubelings and wildling plots, and sagebrush plants were recorded by the same size classes.

We measured density of newly-emerged sagebrush seedlings in all forty-two treatment plots in the first and third summers post-treatment (July/August 2020 and 2022). This was intended to capture the effects of the direct seeding treatment (seeded plots), recruitment from plantings and their offspring (tubeling/wildling plots), and background recruitment unrelated to our treatments (control plots). In each 80 m x 80 m treatment plot, we counted sagebrush seedlings in two 0.25 m² quadrats within each of the four islands (i.e., 8 frames per plot). In tubeling and wildling plots, within each island, one quadrat was placed under the canopy (i.e., adjacent to the stem) of a randomly selected sagebrush, and the other was placed in the interspace between two randomly selected sagebrush plants. In the seeded and control plots, the quadrats were placed at two random points within each of the four islands.

To assess plant community composition in all forty-two plots in the first growing season following project implementation (July/August of 2020), we sampled in the same eight 0.25 m² quadrats per plot where we counted sagebrush seedlings, plus another two quadrats per plot placed approximately 5 m outside of a randomly selected island, in a randomly selected cardinal direction.

### Statistical analysis

To determine the effect of treatment (tubeling vs. wildling) on sagebrush survival (live or dead), we used separate binomial generalized linear mixed models (GLMM) with an observation-level random effect for June 2020, October 2020, and July 2022 data. The seeding treatment, which was established for future comparisons, was not statistically compared to tubeling or wildling survival. We used the same model structure to determine whether the predictor variables that were recorded at the time of planting (planting problems, frost-heaving, physical damage, more than one plant per pot, height) were associated with early tubeling mortality. We ran separate models for each predictor variable, with status of tubelings (live or dead) recorded during October 2020 as the response variable. Five wildling plants were excluded from these and all other analyses as they were determined not to be mountain big sagebrush.

We conducted two separate analyses of sagebrush densities. The first analysis was intended to compare how many new sagebrush had been recruited from seed produced by surviving plantings in tubeling vs. wildling plots over the three years of our study. To do this, we used a negative binomial generalized linear model (GLM) to test for tubeling vs. wildling effects on total numbers of sagebrush, excluding the plantings themselves. We summed across the four belt transects per plot that had been located between planting rows (to exclude originally-planted sagebrush) and across sagebrush size classes. The second analysis compared total sagebrush densities across our three sagebrush establishment methods, but in contrast to the first analysis, it included both new recruits and originally-planted tubelings and wildlings that had survived to year three (2022). Here we used negative binomial GLMMs with an observation-level random effect to test for differences among tubeling, wildling and seeding treatments, with Tukey tests for pairwise comparisons. For individual tubeling and wildling plots, we calculated sagebrush density (#/ha) for each island as the sum of (a) sagebrush densities, summed across size classes, in the belts between planting rows (which excluded plantings) and (b) densities of surviving tubelings/wildlings in the four 225 m^2^ islands per plot. The four island values were then summed to yield a single value per plot. Seeded plot values were total sagebrush densities, summed across size classes, across the four belts per plot (40 m^2^ per plot). For all analyses of sagebrush densities we excluded five plots for which we recorded a sagebrush plant that survived the initial discing/harrowing site preparation (*n* = 4 plants) and/or had an outlying density value, as well as the seven neighboring plots (Fig. S1).

To examine plant community composition across treatment plots we performed non-metric multidimensional scaling (NMDS; R package *vegan* [50]; maximum of 100 random starts, 4 dimensions) on July/Aug 2020 frequency frame data from all islands in the seeded, tubeling and wildling treatments (*n* = 48 islands per treatment). We removed all species that occurred fewer than 10 times across all islands and excluded all unidentifiable species. To examine associations between plant community composition and the establishment of sagebrush from wildling, tubeling and seeding treatments we extracted NMDS axis 1 and 2 scores and used them as predictor variables, with the number of surviving plants (tubeling and wildling) or number of seedlings (seeding) at one-year post-planting (October 2020) for each island as the response variable. We used six separate GLMMs, each with a plot-level random effect, for NMDS1 and NMDS2 tubeling, wildling, and seeding models. We used binomial, beta-binomial, and negative binomial distributions, respectively, for tubeling, wildling, and seeding models.

We used R (version 4.3.3) for all analyses [51]. We used R package *glmmTMB* [52] for statistical models and checked model assumptions (homogeneity of variance, over/under dispersion, outliers, normality) with R package *DHARMa* [53].

### Cost analysis

We recorded the price of our greenhouse-produced sagebrush tubelings, as well as how long it took to plant tubelings, and harvest and plant wildlings. We used those figures, based on a labor wage of 15.00 USD per hour, to calculate the per plant cost of tubelings and wildlings. Then, to calculate the cost per surviving plant of tubelings vs. wildlings, we divided the cost of our planting effort (i.e., 1200 plants * cost per plant) by the number of surviving plants at year 3. Finally, we calculated the cost per established plant for a given treatment by dividing the cost of the planting effort by the estimated total density of sagebrush (i.e., surviving plants plus recruits, the latter originating from seed dispersal of planted). Estimated total density was calculated by multiplying mean sagebrush density at year 3 (regardless of size class) for a given planting type by the total restored area for that planting type (four 15 m x 15 m islands * 12 plots = 10,800 m^2^).

## Results

### Tubeling vs. wildling survival

**Fig. 2:**
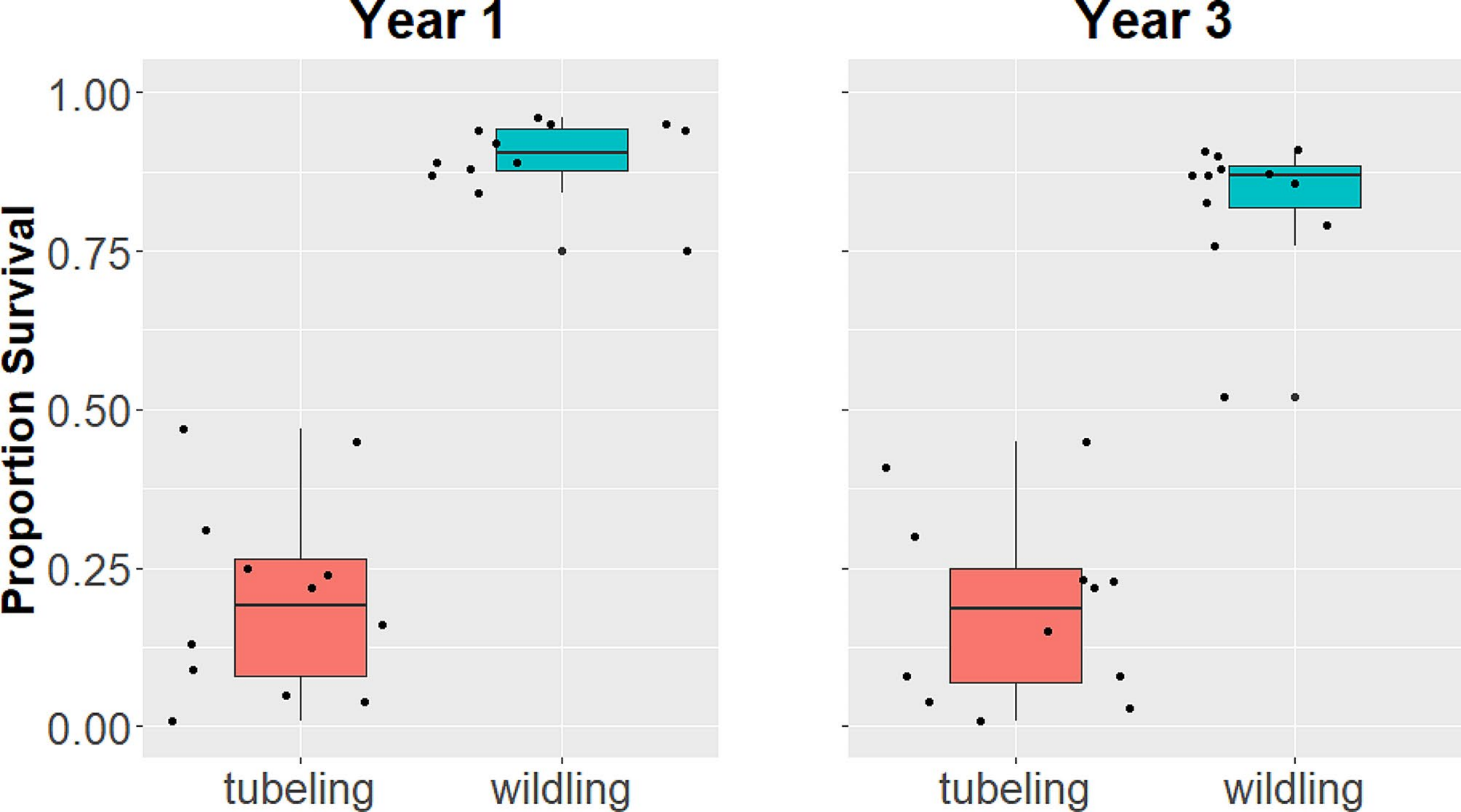
Proportion survival in treatment plots planted with tubeling vs. wildling *Artemisia tridentata* spp. *vaseyana* plants (a) 1 year post-planting (October 2020) and (b) in the third year post-planting (July 2022). Middle line represents the median, box represents interquartile range between 25th and 75th percentiles, and whiskers represent maximum and minimum proportion survival (up to 1.5 of the interquartile range)

Probability of survival was ≥ 4 times higher for wildlings than tubelings from ∼ 6 months post-planting through the third year post-planting (June 2020 χ ^2^ (1) = 111.9, *p* < 0.0001; Oct.2020 χ ^2^ (1) = 114.8, *p* < 0.0001; July 2022 χ ^2^ (1) = 86.12, *p* < 0.0001; Fig. 2, Table S3).

Most mortality occurred over winter between fall planting and the first post-planting census in June 2020. During that census 79% of tubelings and 10% of wildlings had died (Table 1), and mean probability of survival was 92% (± 1 SE = 2.1%) for wildlings, compared to 17% (± 1 SE = 3.7%) for tubelings (Table S3). Only an additional 12 tubelings and 6 wildlings died in the four months between June and October 2020 (Table 1), which encompassed the pronounced summer dry period, and mean probabilities of survival were similar: 91% (± 1 SE = 2.1%) and 16% (± 1 SE = 3.5%), respectively for wildlings and tubelings (Table S3). By the third year post-planting (July 2022) an additional 17 tubelings and 79 wildlings had died (Table 1), and mean probabilities of survival for wildlings and tubelings were, respectively 85% (± 1 SE = 3.2%) and 14% (± 1 SE = 3.7%) (Table S3).

**Table 1:** Number of live and dead *Artemisia tridentata* ssp. *vaseyana* tubelings and wildlings

	# live	# dead since previous census	% survival since previous census
**Tubelings (** ***n*** ** = 1200)**			
June, 2020	254	946	0.21
October, 2020	242	12	0.95
July, 2022	222	17	0.93
**Wildlings (** ***n*** ** = 1195)**			
June, 2020	1079	116	0.90
October, 2020	1073	6	0.99
July, 2022	977	79	0.93

Planting occurred in October/November of 2019. Tubelings and wildlings were censused in the spring/summer (June 2020) and fall (October 2020) following planting, and in the third summer post-planting (July 2022). For the latter census, 3 tubelings and 17 wildlings that had previously been counted as live (in October 2020) were excluded from calculations because the tags used to identify and track plants were missing or in an unexpected location relative to the plant.

### Sagebrush size and reproduction

Live tubelings, measured within five weeks of planting, varied in height from 2 to 23 cm tall with a mean of 12.5 cm (± 1 SE = 0.09) and had a mean crown area of 27.6 cm² (± 1 SE = 0.62; Table S4). The subset of wildlings (*n* = 297) measured within five weeks of planting ranged in height from 6 to 25 cm tall with a mean of 14.3 cm (± 1 SE = 0.21), and a crown area of 35.3 cm² (± 1 SE = 1.29; Table S4). At year three, tubeling and wildling heights were, respectively, 55.0 ± 1.1 mm and 51.9 ± 0.3 mm, and crown areas were 1,893 ± 93 cm² and 3,089 ± 64 cm² (Table S6).

One year following planting, in October 2022, 0.4% of tubelings and 6% of wildings showed evidence of reproduction (presence / absence of flowering stems) (Table S4). By the third year (July 2022), 34% of tubelings and 95% of wildlings showed evidence of reproduction (Table S4).

### Sagebrush densities

**Fig. 3:**
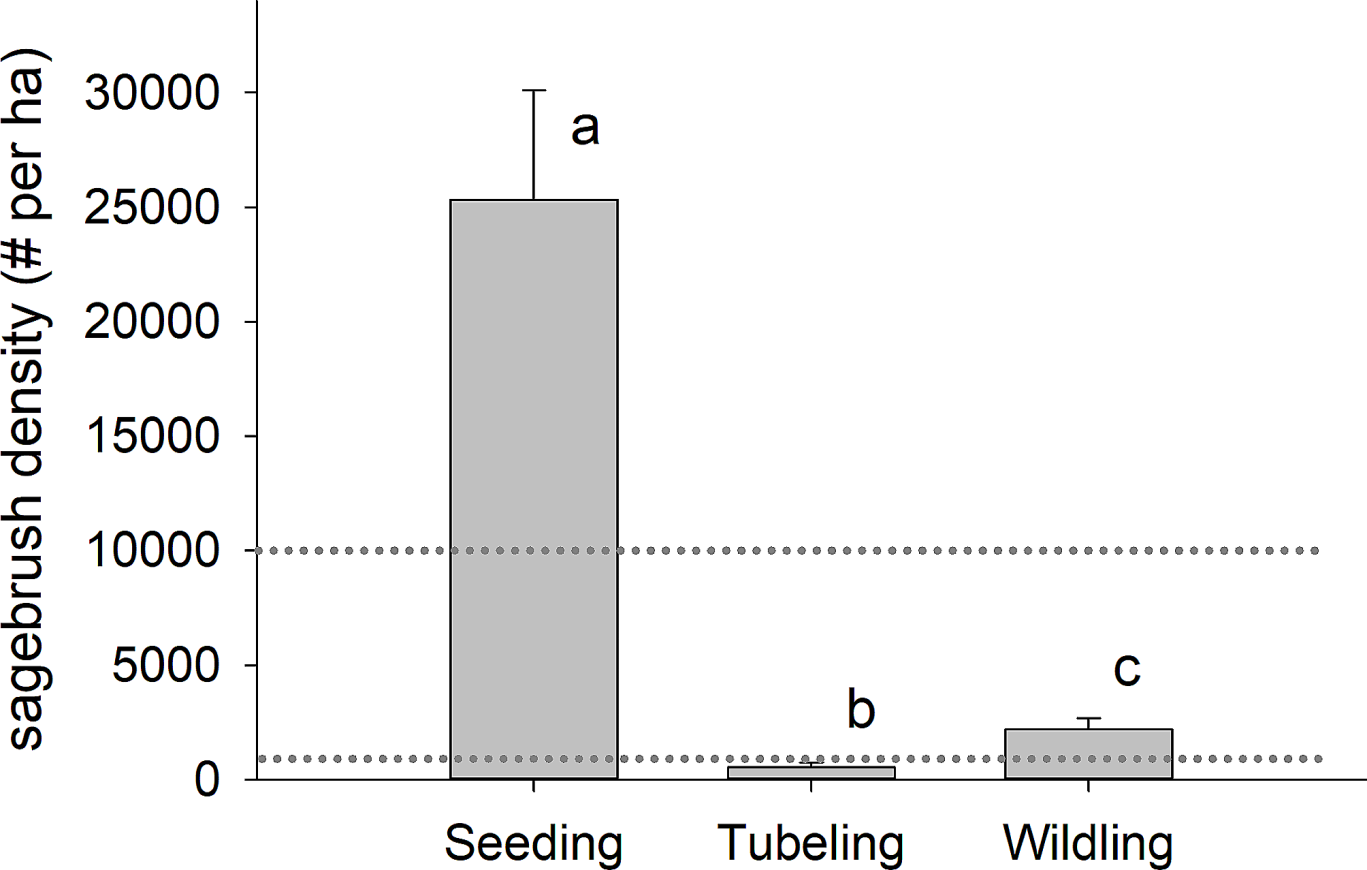
Total densities of *Artemisia tridentata* ssp. *vaseyana* plants across three treatment types in the third year following treatment (July 2022). “Seeding” treatment plots were seeded at a rate of 0.56 kg PLS (pure live seeds) per ha in October 2019. “Tubeling” and “Wildling” plots each were planted at a rate of 1,111 plants per ha in October 2019. The densities presented here include both surviving plantings and presumed recruitment from those plants and their offspring. Shared letters indicate no significant difference at the 0.05 level. Control plots that received no sagebrush seeding or plantings had a mean density of 200 sagebrush per ha, and were not included in statistical analyses. Dotted lines indicate a range of published target densities for sagebrush (see text for details)

In the third year post-planting (July 2022), mean densities of sagebrush (pooled across size classes, excluding newly-established seedlings) in belts between rows of planted tubelings and wildlings did not differ statistically: 392 plants/ha (± 1 SE = 171) and 1,278 plants/ha (± 1 SE = 472), respectively (χ ^2^ (1) = 0.96, *p* = 0.33; Table 2, Table S5). However, mean total densities of sagebrush– including sagebrush in belts between planting rows plus surviving planted tubelings/wildlings– were > 4x higher in wildling than tubeling plots: 2,196 plants/ha (± 1 SE = 485) in wildling plots and 544 plants/ha (± 1 SE = 210) in tubeling plots (Fig. 3; χ ^2^ (2) = 127.47, *p* < 0.0001, Tukey *p* = 0.002). Both were significantly less than mean sagebrush densities in seeded plots: 25,333 plants/ha (± 1 SE = 4,782; χ ^2^ (2) = 127.47, Tukey *p* < 0.0001; Fig. 3, Table S5). Mean density of sagebrush in control plots (that were neither seeded nor planted with tubelings or wildlings) was 200 plants/ha (± 1 SE = 122; Fig. 3).

Assessments of sagebrush seedlings (in 0.25 m^2^ quadrats) indicated that densities in seeded plots were 91–177% greater than in tubeling and wildling plots in July/August 2020. Two years later in July/August 2022, they were 1227–1362% greater (Table S6). In untreated control plots, we found only one sagebrush seedling across all 48 quadrats in the first summer following planting (July/August 2020), and in the third year post-planting (July/August 2022) we found none (Table S6).

**Table 2:** Mean densities, by height class, of *Artemisia tridentata* ssp. *vaseyana* plants recruited from established tubeling or wildling plantings

	Mean (± 1 SE) sagebrush density (#/ha)	
	Tubeling (*n* = 7)	Wildling (*n* = 9)
**Recruits by height class**		
< 15 cm	0 ± 0	28 ± 28
15–50 cm	250 ± 133	917 ± 372
> 50 cm	143 ± 107	333 ± 195
**Recruits (total)**	**392 ± 171**	**1,278 ± 472**

Mean (± 1 SE) densities of *Artemisia tridentata* ssp. *vaseyana* plants, by height class, that were recruited from seed-producing established plants in seven tubeling and nine wildling plots. Data were collected in July 2022 in 1 m x 10 m belts that were oriented to exclude tubelings and wildlings that had been planted in October/November 2019.

### Drivers of sagebrush establishment

We found that 64% of tubelings experienced planting problems that typically contribute to low initial establishment (i.e., roots being exposed or air pockets). These plants had a 12% mean chance of survival in the first year whereas the mean chance of survival for the remaining tubelings without planting problems was nearly twice that (23%; Table S7). No other planting-related factors we assessed in the field were common or significant predictors of first-year tubeling mortality (Table S7).

We found that plant community composition (NMDS axis 1) was a significant predictor of sagebrush plant densities in the tubeling treatment in the first year post-planting (Fig. S3, Stress = 0.19, R^2^ = 0.97; axis 1: χ ^2^ (1) = 4.6, *p* = 0.03; axis 2: χ ^2^ (1) 0.11, *p* = 0.74). Sagebrush survival in tubeling plots was positively related to NMDS Axis 1, as were frequencies of several species in our seed mix that were drill-seeded in fall of 2019 (Fig. S3, Table S8). Conversely, the plant species most negatively related to NMDS axis 1 included the introduced non-native grass *Poa pratensis* L. that was co-dominant prior to implementing our project, as well as two weedy species, *Poa bulbosa* L. and *Alyssum desertorum* Stapf, that also had occurred at high frequencies (Fig. S3, Table S8). Neither NMDS axis 1 nor 2 was a significant predictor of wildling survival (axis 1: χ ^2^ (1) = 3.19, *p* = 0.07; axis 2: χ ^2^ (1) = 2.95, *p* = 0.09) or sagebrush numbers in the seeding treatment (axis 1: χ ^2^ (1) = 1.42, *p* = 0.23, axis 2: χ ^2^ (1) = 0.001, *p* = 0.97).

### Cost analysis

The up-front cost of harvesting and planting a wildling plant was 2-times the cost of purchasing and planting a greenhouse-grown tubeling ($3.00 vs. $1.47 USD; Table S9). The cost per surviving wildling at year 3, however, was less than half the cost per surviving tubeling ($3.68 vs. $7.95 USD; Table S9). When we accounted for both surviving plantings and recruited plants, the cost per established sagebrush plant was $1.07 USD for wildlings and $2.18 USD for tubelings.

## Discussion

There is an increasing need globally to restore degraded dryland ecosystems, but the conditions of these drylands often limit success of traditional restoration methods such as broadscale seeding. Consequently, it is essential to develop innovative approaches, even those that often are considered too costly to be practical. Here we investigated planting containerized seedlings or larger “wildling” transplants to overcome establishment limitations at the seed and early seedling stages [14–18], and in the case of wildlings, demographic limitations at later life stages as well [36]. We found that, based on 3-year survival rates, the per-plant cost of successfully establishing mountain big sagebrush (*A. tridentata* ssp. *vaseyana*) into dryland pasture was 54% less for wildlings, larger plants transplanted with an intact soil-root ball, than for greenhouse grown tubelings. We also found that by the third year post-planting 95% of surviving wildlings and 34% of surviving tubelings showed evidence of reproduction (presence / absence of flowering stems). Both types of plantings recruited new sagebrush plants (2.4 and 3.7 plants, respectively, per surviving wildling/tubeling), illustrating the potential for successfully established plantings to serve as a seed source for natural regeneration within a short (three-year) timeframe. Nonetheless, the cost to establish sagebrush at a given density (including both original plantings and new recruits) was 51% less for wildlings than tubelings due to the high survival of originally-planted wildlings.

### Wildling vs. tubeling performance

We found that, when conditions are appropriate, transplanting mountain big sagebrush wildlings can yield high probability of survival in the first year (91%) and into the third year (85%). These results align with the high (71–90%) first-year survival that has been achieved elsewhere for wildlings of big sagebrush and other shrubs when correct transplanting techniques are used [39, 40, 54]. In contrast, we observed relatively low (16%) first-year survival of mountain big sagebrush tubelings. Other studies have reported low, but also highly variable, first-year survival for sagebrush tubelings [8, 28, 30, 44, 55–59]. Two of these were for the mountain big sagebrush (*A. tridentata* ssp. *vaseyana*) subspecies and reported 13% [59] and 96% [57] survival. Very few of our tubelings died after the first year, but our third-year survival (14%) nonetheless was lower than the one mountain big sagebrush study we found that reported 68% survival at five years post-planting (though planting was combined with herbicide to control weeds [57]). Our 14% third-year survival also was at the lower end of the range reported in a literature review for 5-year survival of the *A. tridentata* ssp. *wyomingensis* subspecies (10 − 74%) [44].

Although our study appears to be the first direct comparison of tubelings vs. wildlings of the mountain big sagebrush subspecies, we found one study that compared tubelings vs. wildlings of the *A. tridentata* ssp. *wyomingensis* subspecies. McAdoo et al. 2013 [42] found that at two years post-planting, at least 3 times as many tubelings than wildlings had survived. But similar patterns were not observed when the authors repeated plantings in a second year. The authors hypothesized that year differences were driven by variability in the quality (i.e., size) of the planting stocks used across the two years. Planting stock may have affected our results as well (see *Factors influencing tubeling and wildling survival* below), but we nonetheless found that planting wildlings yielded very high survival rates under the same conditions that yielded low tubeling survival. Notably, these conditions are in the higher range of elevation and precipitation for big sagebrush, where soil moisture may be sufficient for establishment of larger transplanted wildling plants that, presumably, have higher water demands than do tubelings.

We also compared the potential for seed dispersal from newly-transplanted tubelings vs. wildlings to “nucleate” the landscape (sensu [60]). Although both planting types showed little evidence of reproduction one year post-planting, by the third year most surviving wildlings and more than 1/3 of surviving tubelings had reproduced. Dispersal distances for sagebrush have been documented to range from 1 to 33 m from the mother plant [61], with an estimated maximum of 16 m (on average) [62]. By year three, we found that tubelings and wildlings had both recruited new plants into the 3 m between plantings, and our ongoing work will lend insights into the potential for these recruits to expand the size of islands and/or seed the rest of the landscape. Overall, our results illustrate the value of establishing restoration plantings that can deliver ample seed for major regeneration events when conditions are appropriate for establishment. This is especially relevant for highly episodic species like sagebrush [61, 63]. Accordingly, outcomes of attempts to restore sagebrush from seed are variable, attributable in large part to difficulty in anticipating, and sometimes accessing sites during, appropriate climatic windows [63]. The establishment of plant islands that “nucleate” the landscape has been promoted as a restoration strategy in tropical forests [60], but as far as we know has not been explicitly applied to dryland systems.

### Factors influencing tubeling and wildling survival

Although the planting process itself is known to affect transplant survival [64, 65], we found that wildlings were robust to potential outplanting pitfalls. Conversely, tubelings that were planted well had nearly twice the chance of survival relative to tubelings for which we recorded planting problems (23% vs. 12%). Despite using a reputable contractor and providing explicit directions on how to correctly plant tubelings, in the weeks following planting we found that many tubelings had roots exposed aboveground or large air pockets in the soil around plants. These factors can desiccate roots and prevent uptake of necessary water and nutrients [66]. Transplanting wildlings with an intact soil-root ball may provide them with resistance to these problems because the majority of roots are left intact (though the taproot may be severed) and good root-to-soil contact remains. Similarly, our NMDS results indicate tubelings performed best in the areas of our project site where restoration of the herbaceous community was most successful, perhaps due to the quality of site preparation or to landscape factors. Wildling survival, on the other hand, appeared less sensitive to these factors.

In addition to the physiological and developmental advantages of being transplanted as a large plant with a fully developed, largely intact root system, harvesting our wildlings close to the project site may have been advantageous. Use of local plant materials is a commonly recommended seed-sourcing guideline (e.g., working within seed transfer zones) both for direct seeding and growing greenhouse plants [48, 54]. Local adaptations can result in higher drought or frost resistance, increased growth rates, and higher competitive advantage compared to plants that are not locally-adapted [48]. It is not clear whether collecting seed from 136 km away in a similar elevation and climate yielded tubelings that were locally-adapted. But local sourcing (approximately 11 km away) may have contributed to the high wildling survival we observed. McArthur and Plummer [39] reported a range of 43–100% 1st year survival of mountain big sagebrush wildlings across nine different source populations, and suggested that sourcing wildlings from nearby or climatically-similar populations is crucial in maximizing survival outcomes [39]. In addition to the potential benefits of local adaptation, our locally-collected wildlings may have benefitted from having already faced natural filtering from local environmental and community conditions, perhaps making them even more resilient to environmental stresses occurring at this locale [48]. One disadvantage of our study design is that we were not able to control for genetic effects or local filtering in our comparison of tubelings and wildings. However, choosing between wildlings collected at or near the restoration site vs. greenhouse tubelings grown from seed collected elsewhere constitutes a realistic scenario likely to be encountered by a restoration practitioner.

We suggest three other planting-related factors relevant to survival of tubelings, but less so to wildlings. First, low overall quality of containerized greenhouse stock can reduce survival rates [34, 64]. Our tubelings were grown from seed that was considered by the grower to be “less than ideal” in quality, with only 13% purity, which resulted in a need to re-seed some tubes during the growing process. This meant a portion of the greenhouse stock was younger with less developed roots at the time of planting (Jeff Rebernak, personal communication 2021). This is not uncommon for restoration projects, in which growers are given a limited timeframe to produce plants and often must start growing stock at a time that is out of sync with the natural growing sequence for the species. More closely examining individual traits (e.g., root to shoot ratios) of planting stock could provide insights into less obvious components of tubeling (or wildling) quality and characteristics that might improve restoration outcomes [28, 67, 68]. Second, although quick, common and easy to use, our use of dibbles to create holes for tubelings may have compacted the surrounding soils, which would have resulted in less loose soil to cover the top of the tubelings during planting and later limited root expansion [64]. Third, tubelings were stored outside, on-site, during the 10-day planting period without additional watering, and direct sunlight and exposure to wind could have desiccated and stressed some plants prior to planting [64]. Comparatively, wildlings were planted the same day that they were harvested and the plants were wrapped in burlap during transportation to prevent desiccation.

### Cost-effectiveness of using mountain big sagebrush tubelings and wildlings for restoration

Costs of ecological restoration projects are rarely tracked [69], and when they are, typically are calculated based on metrics such as cost per area, amount of seed distributed, or total planting effort [13]. Here we tracked the cost per established plant to evaluate the efficacy of establishing mountain big sagebrush from tubelings vs. wildlings. We found that the upfront cost of harvesting and planting a sagebrush wildling was more than twice the cost of purchasing a greenhouse-grown tubeling, though the cost difference may have been somewhat inflated because the ground was often frozen in the morning, making it especially hard to harvest wildlings (8 min vs. the 20 s reported in [42]). There also may be ways to reduce costs of tubelings, for example producing greater quantities of stock to reduce “nursery care” [11, 44]. Regardless, because wildling survival was so high, the cost per established (i.e., surviving) wildling plant in the third year was less than 50% of the cost of establishing a tubeling ($3.68 vs. $7.95 USD, respectively). These results underscore the necessity of assessing the “cost as modified by the probability of success” (sensu [23]) to accurately compare restoration methods, particularly in cases (like plantings) where upfront costs are considered high and may deter their use by practitioners. We also went a step further to calculate a different version of cost as modified by the probability of success, using a broader definition of success: the total densities of sagebrush encountered in year 3, the majority of which were recruits from our restoration plantings. Based on these calculations, the cost per established sagebrush was $1.07 USD for wildlings and $2.18 USD for tubelings.

Another consideration for evaluating cost-effectiveness is whether follow-up treatments will be needed. For example, the total sagebrush densities we observed in year 3 post-planting were 544 and 2,196 plants/ha, respectively, for tubelings and wildlings. The latter is greater than the target density of 988 sagebrush plants per ha recommended by the USDA-Natural Resources Conservation Service for big sagebrush (regardless of subspecies) to provide habitat for sagebrush-obligate species [70], but lower than the standards established for mine land reclamation projects (e.g., 10,000 plants per ha for bond release in Wyoming, USA [71]). In contrast, the third, “seeded”, sagebrush treatment that we had established for long-term comparison, clearly far exceeded reasonable restoration targets. The rate at which we seeded (0.56 kg PLS per ha) falls within the recommended range for establishing sagebrush stands, but was intentionally higher than what is commonly used by practitioners since propagule limitation is a concern [71, 72]. Those seeded plots yielded excessively high sagebrush densities (25,333 plants/ha) that would require further management (e.g., mechanical thinning) or an extended timeframe for self-thinning to occur in order to create properly functioning sagebrush stands.

Long-term quality and vigor of plants also should be factored into comparisons of seeding vs. planting of sagebrush. Welch [73] found that, compared to mountain big sagebrush that had established from containerized plantings, plants that had recruited from seed had larger crowns, lower mortality and deeper and more developed root systems, and themselves produced more seeds. This phenomenon has been observed elsewhere for woody species [73–75] and may be because tap root growth is restricted by growth in containers [33, 74]. These benefits of seed-derived plants could also hold true for wildlings, or, alternatively, the soil-root ball with which wildlings are transplanted may effectively act like a “container” once their root growth reaches the extent of the transplanted root ball. Regardless, continued monitoring of our transplanted tubelings and wildlings, as well as the vegetation and soil conditions in all treatment types including seeded plots, will be necessary to fully evaluate the cost-effectiveness and overall success of the three sagebrush establishment methods.

### Management implications

Matching plant establishment methods and plant materials to environmental conditions, as well as working within the bounds of resources available for a project, are important factors in the success or failure of any restoration project. While some of these variables often are out of the control of practitioners, when possible, they should nonetheless be taken into consideration to increase the likelihood of success. We offer the following considerations for choosing among plant establishment methods:

1. Project scale is a key factor in selecting a plant establishment approach. Seeding is likely most appropriate for projects that must be implemented in a restricted time-frame or over a large area. Wildlings or tubelings, on the other hand, would be more appropriate for multi-phase projects in which smaller sub-areas are treated in successive years, as a complement to seeding treatments, or for the establishment of restoration islands that are meant to nucleate the landscape (sensu [60, 76]).
2. To use wildlings for large projects, having a sagebrush stand near the target field site will streamline the harvesting and planting process and enable sourcing from locally-adapted populations. In our case, the donor site was slated for future vegetation removal, but in situations where this is not the case, it is necessary to ensure that harvesting wildlings can be done sustainably and in a way that does not negatively affect the donor site. Additionally, in some cases, permitting or other permissions may be required.
3. The decision of when to harvest and plant wildlings, plant tubelings or perform seeding should be dependent upon anticipated precipitation and soil moisture at the harvest and target locations. Successful seedings typically occur in areas that receive more than 30.5 cm of mean annual precipitation [24] and can occur in late fall or early winter. In contrast, tubeling and wildling plantings in the Intermountain West should take place when temperatures and risk of frost heaving are low and soil moisture and chance of precipitation are high [48, 63, 77]. McAdoo et al. (2013) also hypothesized that dry soil conditions at the time of harvest led to increased root tearing of wildlings. In our study, the high elevation study site with relatively high precipitation likely contributed to successful establishment and subsequent reproduction by plantings.
4. Our results support the well-established recommendation that suppressing perennial grass competition prior to planting shrub species improves shrub survival [42].

## Conclusions

Our results indicate the use of transplanted “wildlings” as a promising strategy for restoring mountain big sagebrush. We found that transplanting wildlings yielded very high survival (85%) throughout the third-year post-planting. Wildling plants that have developed root systems (that remain largely intact during the transplanting process) and have already faced various environmental filters, are a potential avenue for achieving high early establishment rates in a region where moisture often limits plant establishment in early growth stages. Survival of “tubeling” transplants, on the other hand, was much lower (14%), with most mortality occurring in the 1st year.

In order to determine which method of establishing plants is appropriate for a project, land managers must weigh the potential success of each method against several practical considerations (e.g., scale, labor, feasibility of site preparation and treatment implementation; Fig. S4). Tracking project costs, which often are not reported alongside scientific evaluations of alternative restoration treatments, allowed us to calculate the per-plant cost of establishing a sagebrush plant from tubeling vs. wildling. Despite upfront costs of transplanting wildlings being more than double that of tubelings, because of high wildling survival, we found the per-plant cost of establishing a sagebrush wildling to be less than 50% of the cost of establishing a sagebrush tubeling. Our results illustrate how these types of calculations can illuminate clear trade-offs among alternative approaches to establishing plants in restoration settings.

Our results also illustrate the potential for dryland restoration plantings to “nucleate” the landscape via natural regeneration during ideal climate windows (*sensu* [76]). Despite much lower survival and fewer numbers of reproductive plants than wildlings, tubelings nonetheless recruited plants and increased overall sagebrush densities on the landscape. Given the severe establishment bottlenecks encountered by plants in dryland settings [14–18], we conclude that transplantation of larger “wildlings” of woody species that may yield very high survival rates, along with the potential for plantings to ‘nucleate’ the landscape (both wildlings and tubelings in our case), hold promise for improving restoration outcomes.

### Electronic Supplementary Material

Below is the link to the electronic supplementary material.

Supplementary Material 1

## Data Availability

The data that support the findings of this study are available in Figshare with the identifier: https://10.6084/m9.figshare.25236892.
