# Supplementary material for: Transplanted sagebrush “wildlings” exhibit higher survival than greenhouse-grown tubelings yet both recruit new plants
Source: BMC Ecol Evol. 2024 Apr 22;24:50. doi: 10.1186/s12862-024-02236-z (PMC11034100; doi:10.1186/s12862-024-02236-z)
Supplement: Supplementary file 1 — Supplementary Material 1 [file 12862_2024_2236_MOESM1_ESM.pdf]

Supplementary material. *Bailey et al. Transplanted sagebrush "wildlings" exhibit higher survival than greenhouse-grown tubelings yet both recruit new plants*

Table S1. Monthly precipitation (mm) for one month prior to planting (September 2019) through the final sampling (August 2022). Bottom row is 30-yr (1991-2020) monthly means. Bold indicates values that were > 2x the 30-yr monthly mean.

	Jan	Feb	Mar	Apr	May	Jun	Jul	Aug	Sep	Oct	Nov	Dec
2019									**104**	53	28	46
2020	97	61	86	64	48	91	15	3	15	25	76	61
2021	46	104	30	36	69	0	**48**	53	41	**137**	33	119
2022	66	13	18	43	69	41	5	61				
30-yr mean	80	63	61	64	69	47	20	34	45	57	70	82

Table S2. Twenty-two plant species included in herbaceous seed mix, including whether they are native to the area (Nativity status), where seed was sourced (State of origin), and the rate (kilograms of pure live seed per hectare) at which it was applied.

	**Scientific name**	**Common name**	**Nativity status**	**State of origin**	**kg pls/ha**
**Perennial grasses**	*Achnatherum lettermanii*	Letterman's needlegrass	Native	MT	0.56
	Bromus marginatus	Mountain brome	Native	ID	1.12
	Festuca idahoensis	Idaho fescue	Native	ID	0.56
	Festuca ovina	Sheep fescue	Non-native	OR	1.40
	Leymus cinereus	Basin wildrye	Native	ID	0.22
	Pascopyrum smithii	Western wheatgrass	Native	ID	1.68
	Poa secunda	Sandberg bluegrass	Native	WA	0.28
	Pseudoroegneria spicata	Bluebunch wheatgrass	Native	ID	2.02
**Perennial forb / subshrub**	*Achillea millefolium*	Western yarrow	Native	ID	0.11
	Astragalus cicer	Chickpea milkvetch	Non-native	MT	0.84
	Cleome serrulata	Rocky Mountain beeplant	Native	UT	0.95
	Gaillardia aristata	Blanketflower	Native	OR	0.28
	Linum perenne	Blue flax	Non-native	ID	0.11
	Lomatium dissectum	Fernleaf biscuitroot	Native	UT	0.11
	Lomatium triternatum	Nineleaf biscuitroot	Native	UT	0.22

	Lupinus sericeus	Silky lupine	Native	UT	0.11
	Medicago sativa	Alfalfa	Non-native	MT	1.12
	Onobrychis viciifolia	Sainfoin	Non-native	ID	2.24
	Penstemon strictus	Rocky Mountain penstemon	Native	UT	0.11
	Ratibida columnifera	Upright prairie coneflower	Native	OR	0.11
	Solidago canadensis	Canada goldenrod	Native	ID	0.11
	Sphaeralcea coccinea	Scarlet globemallow	Native	UT	0.11

Table S3. Estimated marginal means from generalized linear mixed models for tubeling and wildling survival data for June 2020, October 2020 and July 2022.

**Means estimate on original proportion scale (DF = 21)**				
**Treatment**	**Probability**	**SE**	**Lower CL**	**Upper CL**
**June 2020**				
Tubeling	0.171	0.037	0.107	0.262
Wildling	0.917	0.021	0.863	0.951
**October 2020**				
Tubeling	0.161	0.035	0.100	0.247
Wildling	0.911	0.021	0.855	0.946
**July 2022**				
Tubeling	0.143	0.037	0.087	0.226
Wildling	0.847	0.032	0.761	0.905

Table S4. Traits of tubelings and wildlings planted in October 2019 within 5 weeks of planting, the summer following planting (June 2020), one-year post-planting (October 2020), and in the third year post-planting (July 2022). Means ± 1 SE are displayed for the two continuous variables, height, and crown area (calculated from longest axis of canopy and its perpendicular). The remaining are ordinal (yes/no) variables. Large herbivore damage is excluded from the table because none was observed. See Table 1 for sample sizes.

Trait	Oct / Nov 2019		Jun 2020		Oct 2020		Jul 2022	
	Tubeling	Wildling	Tubeling	Wildling	Tubeling	Wildling	Tubeling	Wildling
Height	12.5 ± 0.09 cm	14.3 ± 0.21 cm	-	21.4 ± 0.16 cm *	-	-	55.0 ± 1.06cm	51.9± 0.33cm
Crown Area	27.7 ± 0.64 cm^2	35.0 ± 1.28 cm^2	-	129.3 ± 2.43 cm^2 *	-	-	1893± 92.78cm^2	3088 ± 64.19 cm^2
% Reprod-uctive	0%	0%	0%	0%	0.40%	6%	34%	95%
Insect Damage*	-	13%	0%	28%	0.1%	17%	5%	10%
Rodent Damage*	-	-	3%	3%	0%	0%	-	-

* Denotes percentage of all plants (dead or live), otherwise percentage is of live plants only.

Table S5. Estimated marginal means on model scale from analyses of sagebrush recruitment densities (measured in belts between plantings) and total sagebrush densities (including plant measured in belts plus surviving plantings).

Treatment	emmean	SE	Lower CL	Upper CL
**Recruitment**				
Tubeling	0.89	0.558	-0.205	1.98
Wildling	1.49	0.401	0.704	2.28
**Total densities**				
Seeding	7.61	0.163	7.30	7.93
Tubeling	3.72	0.396	2.94	4.49
Wildling	5.20	0.224	4.76	5.64

Table S6. Densities of newly-emerged sagebrush seedlings in seeded, tubeling, wildling and control plots. Treatments were implemented in October/November 2019, and densities were assessed in ten 0.5 m x 0.5 m quadrats per plot.

	Sagebrush seedling density (#/m²)			
**Date**	**Seeded (n=12)**	**Tubeling (n=12)**	**Wildling (n=12)**	**Control (n=6)**
July/Aug 2020	12.6 ± 2.7	0.06 ± 0.06	0.05 ± 0.05	0 ± 0
July/Aug 2022	5.3 ± 1.1	0.06 ± 0.06	0.18 ± 0.1	0 ± 0

Table S7. Estimated marginal means from binomial generalized linear mixed models examining possible associations between variables and 1-year tubeling mortality (* indicates statistical significance at the 0.05 level).

	Means estimate on proportion scale (DF = 1197)					Statistical results	
**Covariate (% tubeling/wildling)**	**Level**	**Estimate**	**SE**	**Lower CL**	**Upper CL**	**Chi-square (df = 1)**	**p**
Planting problems (64/2) *	Absent (0)	0.227	0.558	0.136	0.354	20.6	$5.7 * 10^{-06}$
	Present (1)	0.119	0.033	0.068	0.199		
Frost Heaved (19/0)	Absent (0)	0.153	0.044	0.085	0.260	0.099	0.9
	Present (1)	0.162	0.051	0.084	0.289		
More Than One (8/6)	Absent (0)	0.151	0.042	0.085	0.254	2.105	0.147
	Present (1)	0.203	0.065	0.104	0.359		
Physical Damage (10/1)	Absent (0)	0.151	0.043	0.084	0.256	1.256	0.263
	Present (1)	0.192	0.063	0.097	0.345		
	**Means estimate on logit scale**					**z-value**	**p**
Height	-	-0.009	0.027	-		-0.319	0.750

Table S8. Frequency of all species recorded in 0.25m^2 frequency frame sampling of project area (n = 420) and adjacent untreated reference area (n = 60) in the first growing season following project implementation, July/August 2020. Species included in seed mix are bolded. * constitute groupings of several species that were unidentifiable at the time of sampling because plants were newly established and did not have any reproductive features.

Species code	Scientific name	Average frequency %	Standard error ±
**Project area**			
THAR	*Thlaspi arvense*	97.6	0.82
PODO	*Polygonum douglasii*	92.6	1.41
COTI	*Collomia tinctoria*	91.7	2.01
TAOF	*Taraxacum officinale*	65.0	4.51
**POSE**	***Poa secunda***	57.9	3.88
UG	*Unidentified grass*	43.3	4.45
**ACMI**	***Achillea millefolium***	42.6	3.49
VEPE	*Veronica peregrina*	41.9	4.75
POBU	*Poa bulbosa*	38.8	4.70
UF	*Unidentified forb*	36.0	4.20
BRIN	*Bromus inermis*	31.4	2.65
**MESA**	***Medicago sativa***	29.3	3.00
COLI	*Collomia linearis*	28.6	4.21
**Lupine**	***Lupine spp.***	27.9	3.08
DRVE	*Draba verna*	27.4	3.56
CHAL	*Chenopodium album*	24.8	2.98
ELTR	*Elymus trachycaulus*	23.3	3.12
MELU	*Medicago lupulina*	23.1	3.00
**FEID**	***Festuca idahoensis***	22.1	3.30
ARTR	*Artemisia tridentata*	21.0	4.43
RATE	*Ranunculus testiculatus*	20.2	4.05
ACHY	*Achnatherum hymenoides*	16.2	3.30
**ONVI**	***Onobrychis viciifolia***	15.5	1.71
ALDE	*Alyssum desertorum*	15.5	3.13
Elymus	*Elymus spp.*	14.5	2.32
POAV	*Polygonum aviculare*	13.8	2.15
Hedysarum	*Hedysarum spp.*	13.8	2.34
CIAR	*Cirsium arvense*	13.8	1.96
**PEST**	***Penstemon strictus***	12.9	1.75
**PSSP**	***Pseudoroegneria spicata***	11.4	2.56
LASE	*Lactuca serriola*	11.0	1.98

**LIPE**	***Linum perenne***	10.7	1.58
ALPR	*Alopecurus pratensis*	10.0	1.74
ELRE	*Elymus repens*	9.3	2.63
TRDU	*Tragopogon dubius*	8.3	1.40
**RACO**	***Ratibida columnifera***	8.3	1.44
CHBO	*Chenopodium botrys*	8.3	2.07
Festuca	*Festuca spp.*	7.9	2.14
POPR	*Poa pratensis*	7.4	1.81
Sporobolus	*Sporobolus spp.*	6.4	1.66
GARA	*Gayophytum ramosissimum*	5.7	2.02
Bromus	*Bromus spp.*	5.0	1.46
HECO	*Hesperostipa comata*	5.0	2.19
ANPA	*Antennaria parvifolia*	4.5	0.91
NEBR	*Nemophila breviflora*	4.5	2.51
ARLU	*Artemisia ludoviciana*	4.0	1.08
Poa	*Poa spp.*	4.0	1.32
SYAS	*Symphyotrichum ascendens*	3.6	1.56
VETH	*Verbascum thapsus*	3.6	1.61
CABU	*Capsella bursa-pastoris*	2.9	0.85
Oenothera	*Oenothera spp.*	2.1	1.39
LIVU	*Linaria vulgaris*	2.1	0.94
CHLE	*Chenopodium leptophyllum*	1.9	0.98
VIAM	*Vicia americana*	1.7	0.59
Arabis	*Arabis spp.*	1.2	0.78
**CLSE**	***Cleome serrulata***	1.2	0.61
MAMA	*Matricaria matricarioides*	1.2	0.61
EREA	*Erigeron eatonii*	1.0	0.46
Rumex	*Rumex spp.*	1.0	0.75
ASMI	*Astragalus miser*	0.7	0.53
Cryptantha	*Cryptantha spp.*	0.7	0.53
Lotus	*Lotus spp.*	0.7	0.53
NA(BR)	*Navarretia (breweri)*	0.7	0.40
VIPU	*Viola purpurea*	0.7	0.53
ASCO	*Astragalus convallarius*	0.5	0.48
Claytonia	*Claytonia spp.*	0.5	0.33
Collinsia	*Collinsia spp.*	0.5	0.33
EQLA	*Equisetum laevigatum*	0.5	0.48
**LOTR**	***Lomatium triternatum***	0.5	0.33
Symphyotrichum	*Symphyotrichum spp.*	0.5	0.33
Aster	*Aster spp.*	0.2	0.24

Cirsium	*Cirsium spp.*	0.2	0.24
COPA	*Collinsia parviflora*	0.2	0.24
Crepis	*Crepis spp.*	0.2	0.24
DISP	*Distichlis spicata*	0.2	0.24
Delphinium	*Delphinium spp.*	0.2	0.24
HOJA	*Hordeum jubatum*	0.2	0.24
LAOC	*Lappula occidentalis*	0.2	0.24
PHPR	*Phleum pratense*	0.2	0.24
SPCR	*Sporobolus cryptandrus*	0.2	0.24
Trifolium	*Trifolium spp.*	0.2	0.24
**Reference area**			
BRIN	*Bromus inermis*	98.3	0.02
POPR	*Poa pratensis*	75.0	0.12
TAOF	*Taraxacum officinale*	66.7	0.12
COTI	*Collomia tinctoria*	48.3	0.14
Lupine	*Lupine spp.*	46.7	0.15
ALPR	*Alopecurus pratensis*	45.0	0.09
POBU	*Poa bulbosa*	35.0	0.13
PHPR	*Phleum pratense*	23.3	0.12
PODO	*Polygonum douglasii*	23.3	0.11
ALDE	*Alyssum desertorum*	20.0	0.08
ACMI	*Achillea millefolium*	20.0	0.11
SYAS	*Symphyotrichum ascendens*	15.0	0.07
TRDU	*Tragopogon dubius*	15.0	0.04
CIAR	*Cirsium arvense*	13.3	0.10
ARTR	*Artemisia tridentata*	10.0	0.05
VEPE	*Veronica peregrina*	6.7	0.03
LASE	*Lactuca serriola*	3.3	0.02
UF	*Unidentified forb*	3.3	0.02
AGUR	*Agastache urticifolia*	1.7	0.02
COLI	*Collomia linearis*	1.7	0.02
GARA	*Gayophytum ramosissimum*	1.7	0.02
HEOC	*Hedysarum occidentale*	1.7	0.02
LAOC	*Lappula occidentalis*	1.7	0.02
LIVU	*Linaria vulgaris*	1.7	0.02
MELU	*Medicago lupulina*	1.7	0.02
ORLU	*Orthocarpus luteus*	1.7	0.02

Table S9. Comparison of estimated costs (in USD) for restoring sagebrush from tubeling vs. wildling sagebrush plants at Fox Hills Ranch. "Cost per plant" reflects the cost of obtaining and planting a single tubeling or wildling. Tubelings were purchased from a greenhouse, whereas we harvested wildlings from a nearby site, which took 8 min (0.133 hr) per plant. We estimated that planting took 30 sec (0.008 hr) per tubeling and 4 minutes (0.067 hr) per wildling. We used a $15/hr labor wage. "Cost per surviving planting" reflects the cost of planting 1200 tubelings and wildlings relative to the number of surviving plants in year 3. "Cost per established plant" reflects the cost of planting 1200 tubelings and wildlings relative to all established sagebrush plants in year 3. This latter figure included both surviving plantings and new recruits ("# surviving plants + recruits") and was calculated according to mean densities across treatment plots and the total area planted with tubelings and wildlings (four 15m x 15m islands * 12 plots for each planting type).

		**Tubeling**	**Wildling**
**Cost per plant**	Greenhouse cost	$1.35	--
	Labor cost: Harvest	--	0.133 hr* $15.00/hr = $1.99
	Labor cost: Planting	0.008 hr * $15.00/hr = $0.12	0.067 hr *$15.00/hr = $1.01
	**Cost per plant**	**$1.47**	**$3.00**
**Cost per surviving plant**	Cost of 1200 plantings	1200*$1.47 = $1,764	1200*$3.00 = $3,600
	Number of surviving plants (Year 3)	222	977
	**Cost per surviving planting**	$1,764/222 plants = **$7.95**	$3,600/977 plants = **$3.68**
**Cost per established plant**	# surviving plants + recruits	809	3,349
	**Cost per established plant (including recruits)**	$1,764/809 plants = **$2.18**	$3,600/3,349 plants = **$1.07**
**Excluded costs**	Associated costs excluded from analysis	Seed testing and cleaning, transportation from greenhouse to site, planting tools	Permit for harvesting, burlap, twine, planting tools

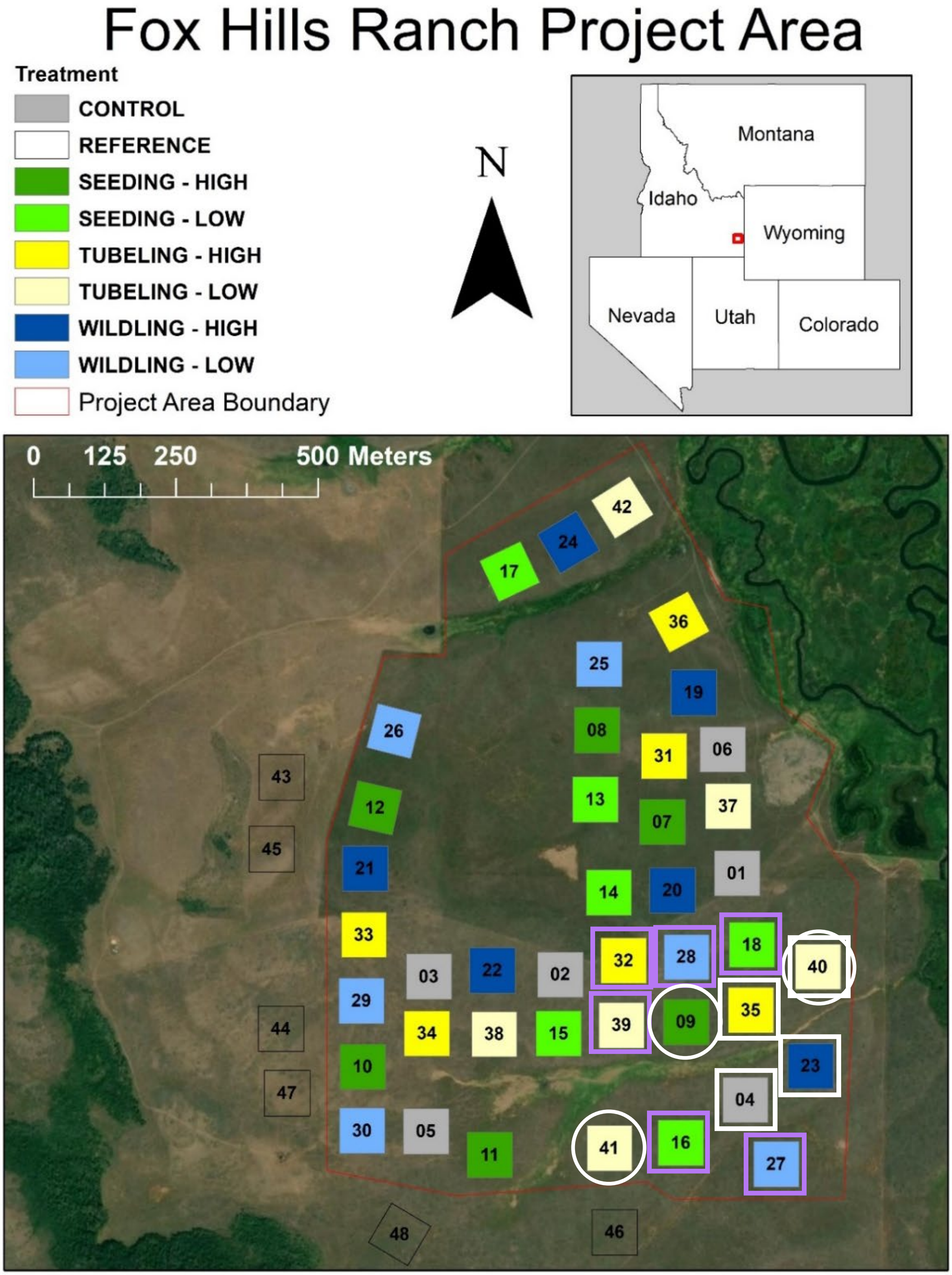

Sources: Esri, Maxar, Earthstar Geographics, HERE, Garmin, © OpenStreetMap contributors, and the GIS User Community. Powered by Esri.

Figure S1. Research plot layout within project area at Fox Hills Ranch in Southeastern Idaho. Colored boxes (1-42) are 80 m x 80 m plots within the project area that were disced/harrowed/herbicided/seeded before applying sagebrush establishment treatments, and clear boxes (43-46) are untreated reference areas outside the project area. “High” and “Low” in the legend refer to arrange of the four sagebrush islands per plot in high vs low densities (see Fig. 1). The 80 m x 80 m plots were located a minimum of 15 m from each other. Four sagebrush plants that survived the initial discing/harrowing during site preparation were found across the three plots circled in white. Those plots were excluded from analyses of sagebrush recruitment, as were plots with white outlines that had a sagebrush density value that was the highest for its treatment. Plots with purple outlines were excluded due to their close proximity.

References:

1. Esri. "Imagery" [basemap]. Scale Not Given."World Imagery". June 13, 2013. https://services.arcgisonline.com/ArcGIS/rest/services/World_Topo_Map/MapServer (March 13, 2021).
2. Esri. "Light Gray Canvas" [basemap]. Scale Not Given. "World Light Gray Base". September 16, 2011. https://services.arcgisonline.com/ArcGIS/rest/services/Canvas/World_Light_Gray_Base/MapServer (March 13, 2021).

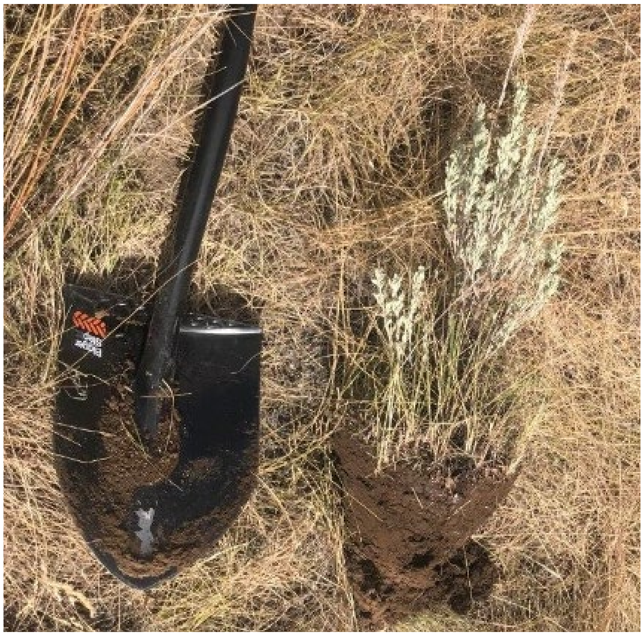

Figure S2. “Wildling” sagebrush plant harvested with soil-root ball intact.

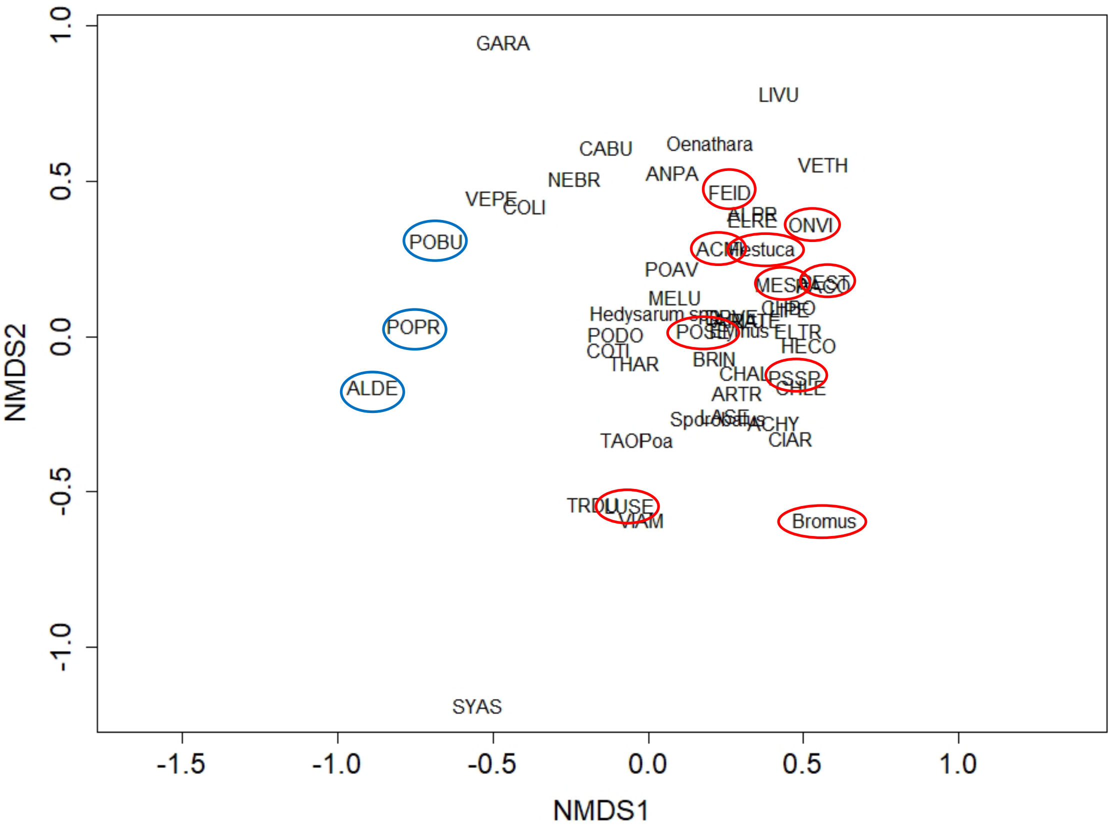

Figure S3. Plant species scores along NMDS axes 1 and 2 based on frequency frame data in the project area (42 plots with ten 0.25 m^2 frames per plot) in July/Aug 2020. Species circled in red were included in the seed mix that was drill-seeded in fall 2019 (see Table S7 for species codes). Species circled in blue include the introduced grass *Poa pratensis* (POPR), as well as weedy species *Alyssum desertorum* (ALDE) and *Poa bulbosa* (POBU), all of which occurred in high frequencies in the untreated reference area and in the treated project area prior to implementation.

TREATMENT	SEEDING	TUBELINGS	WILDLINGS
DECISION-MAKING QUESTIONS	• Can local seed be harvested?<br>• How will seed be stored?<br>• When will seeding be done?<br>• What is method of seeding?<br>• How can seed to soil contact be increased (i.e. imprinter, roller)?	• What seed will be used for plants?<br>• Can quality plants be produced?<br>• Who will be planting?<br>• What planting tools are appropriate?	• Is there an intact stand nearby?<br>• What permits / permissions are needed to harvest wildlings?<br>• What is the distance from harvest stand to field site?<br>• Who will be planting?
RELATIVE INTENSITY OF ACTIVITIES	**PREPARATION**<br>Purchase or collect seed, seed cleaning, seed testing, seedbed preparation, herbicide application<br>**IMPLEMENTATION**<br>Broadcast seeding	**PREPARATION**<br>Purchase seed (clean & test), contract with greenhouse, seedbed preparation, herbicide application<br>**TRANSPORTATION**<br>From greenhouse to field site<br>**IMPLEMENTATION**<br>Outplanting into field site	**PREPARATION**<br>Find harvest stand, obtain permits or permissions for harvest, seedbed preparation, herbicide application<br>**IMPLEMENTATION**<br>Harvesting from nearby stand<br>**TRANSPORTATION**<br>From harvest stand to field site<br>**IMPLEMENTATION**<br>Outplanting into field site
ASSOCIATED COSTS	Seedbed preparation, herbicide application, Seed (labor & materials if harvesting locally), seed cleaning, seed testing, labor & equipment for seeding	Seedbed preparation, herbicide, seed (labor & materials if harvesting locally), greenhouse care (containers, soil, daily watering), transportation from greenhouse to site, labor for planting, tools for planting	Seedbed preparation, herbicide, harvest permit, burlap, twine, shovels, transportation, labor for harvest and planting

Figure S4. Comparison of factors influencing choice of sagebrush establishment approach based on a mountain big sagebrush restoration site in southeastern ID.
